# Pyridoxine-dependent and other pyridoxine-responsive epilepsies—insights into phenotype overlapping and the long-term outcome in a cohort of 21 children

**DOI:** 10.3389/fneur.2026.1855962

**Published:** 2026-07-13

**Authors:** Ružica Kravljanac, Biljana Vučetić Tadić, Vladimir Oparnica

**Affiliations:** Faculty of Medicine, Institute for Mother and Child Healthcare of Serbia, University of Belgrade, Beograd, Serbia

**Keywords:** ALDH7A1, epilepsy, PLPBP, PNPO, pyridoxine dependent, pyridoxine responsive

## Abstract

**Aim:**

Evaluation of etiology, phenotype, and long-term outcome, and defining the predictors of outcome in children with pyridoxine-responsive seizures.

**Methods:**

The study included all children with pyridoxine-responsive seizures treated in our hospital from 2006 to 2025. Serial video electroencephalography, brain MRI, metabolic, genetic analyses, and psychological assessment were done in all cases. All patients were divided into two groups: patients with pyridoxine-dependent epilepsy (PDE) associated with pathogenic variants in *ALDH7A1, PNPO, and PLPBP* (I group) and patients with pyridoxine-responsive epilepsy (PRE) due to other etiologies (II group). The early-onset seizures were initially treated by antiseizure medication (ASM), and if at least two of the ASMs failed to stop the seizures, pyridoxine was given (100 mg/day, iv). Analyzed parameters were age of seizure onset, period from seizure onset to pyridoxine introduction, brain MRI, type of seizures, etiology, ASM, and predictors for outcome. The outcome included seizure control and neurological development. Mann–Whitney test, Fisher’s exact test, and Firth penalized logistic regression were used to test for statistical significance.

**Results:**

Twenty-one patients were included: 10 in group I and 11 in group II with various etiologies. Median age at seizure onset in both cohorts was 2 (range 1–11) days. Mann–Whitney U test demonstrated no significant difference between the two groups (*p* = 0.79). The median time from seizure onset to pyridoxine treatment was 12 days in PDE (range 1–61 days) and 10 days (range 1–98) in PRE, and the difference was not statistically significant (*p* = 0.65). Developmental delay was present in 6/10 (60%) PDE patients and 5/11 (45.5%) PRE patients. Seizure freedom was attained in 8/10 (80%) PDE and 8/11 (72.7%) PRE patients. MRI abnormalities were seen in 8/10 (80%) PDE and 7/11 (63.6%) PRE patients. The difference in these parameters between the two groups was significant.

**Discussion:**

Etiologic heterogeneity and phenotype overlapping between PDE and PRE, atypical presentations, and good initial response to pyridoxine regardless of genetic and neuroimaging findings suggest the introduction of pyridoxine in all infants when two ASMs have failed. Despite appropriate pyridoxine treatment, more than half of patients with PDE had developmental delay and resistant seizures, suggesting the complexity of the underlying mechanisms.

## 1 Introduction

Pyridoxine-responsive seizures (PRS) are characterized by early-onset seizures and epileptic encephalopathy, which respond to pyridoxine. This entity includes a wide spectrum of etiologies beyond known genetic disorders, which cause pyridoxine-dependent epilepsy. Pyridoxine-dependent epilepsy (PDE) is caused by several independent genetic disorders which interfere with the bioavailability of pyridoxine and pyridoxal-phosphate: antiquitin deficiency (due to pathogenic *ALDH7A1* variants), hypophosphatasia, hyperphosphatasia, pyridox(am)ine 5′-phosphate oxidase (PNPO) deficiency, and pyridoxal-phosphate (PLP) binding protein deficiency (formerly called PROSC deficiency, and now PLPBP) (1, 2).

The most frequent cause of PDE is due to antiquitin deficiency (pathogenic ALDH7A1 variants) involved in lysine catabolism (3). ALDH7A1 encodes *α*-aminoadipic semialdehyde (α-AASA) dehydrogenase, and its deficiency causes accumulation of α-AASA, Δ1- piperideine-6-carboxylate (P6C), and pipecolic acid (PA). Piperideine-6-carboxylate inactivates pyridoxal-5′-phosphate, which is involved as a cofactor in more than 70 enzymatic reactions in human metabolism (4, 5). Most of the proposed pathogenic mechanisms of seizures and developmental delays in PDE-ALDH7A1 are related to pyridoxal-5′-phosphate deficiency (6–8). Impaired mitochondrial electron transport chain functions and abnormal tricarboxylic acid cycle (TCA) metabolites were described in a patient with PDE-ALDH7A1 and in zebrafish models (9). Ambrose et al. reported the improvements in neurodevelopmental outcome in a patient with PDE-ALDH7A1 treated with triheptanoin. They found a mild complex I deficiency in muscles and suggested that PDE-ALDH7A1 is associated with mitochondrial electron transport chain dysfunction and deficiencies in the energy production pathways. Anaplerotic therapy, such as triheptanoin, which provides ketones and substrates to the TCA cycle, might be helpful in improving neurodevelopmental outcomes of patients with PDE-ALDH7A1 (10). According to the supposed biochemical base of disorder, early introduction of a lysine-restricted diet with enrichment of arginine, which inhibits lysine transport, might be effective for cognitive outcomes (11, 12). Schuurmans et al. highlight the therapeutic potential of antisense oligonucleotide therapy targeting AASS to reduce neurotoxic metabolite accumulation in PDE (13).

According to Mills et al. (5), suggestive clinical features for PDE include seizures in infancy without brain malformation or injury; cryptogenic seizures in a normal infant with normal perinatal history; in phenotype in neonates suggestive for hypoxic–ischemic encephalopathy and with difficult-to-control seizures; the occurrence of long-lasting focal or unilateral seizures; resistance to ASMs; and seizures that are partially responsive to ASMs, associated with developmental delay and intellectual disability in neonates and infants with encephalopathy, irritability, restlessness, abnormal crying, and vomiting and with positive response to pyridoxine (5). The classic form of PDE associated with pathogenic variants in *ALDH7A1* is characterized by seizure onset within the first few weeks of life, “dramatic” presentation of prolonged seizures and status epilepticus in the form of focal, generalized, atonic seizures, myoclonic events, even infantile spasms, and sometimes with electrographic seizures without clinical correlates. Atypical PDE-ALDH7A1 is characterized by late-onset seizures between late infancy and 3 years, seizures that initially respond to ASM and then become intractable, seizures during early life non-responding to pyridoxine but with subsequent control with pyridoxine several months later and prolonged seizure-free intervals (≤5 months) after discontinuation of pyridoxine (14).

The less frequent PDE type is due to the PLP binding protein deficiency associated with pathogenic variants in the PLPBP gene. Recently published multicenter case series and systematic review collected a total of 54 individuals with PLPBP variants. According to the degree of neurodevelopmental impairment and seizure control, more than two-thirds of patients had moderate to severe disease, with seizure onset within the first week of life. Antenatal anomalies, prematurity, fetal distress, microcephaly, and brain MRI abnormalities (65%) are common (15).

In the classical form of PDE type due to pyridoxal 5′- phosphate (PLP) binding protein deficiency, the most common features are: seizure onset within the first month of life associated with encephalopathy (inconsolable crying, hyperalertness, jitteriness, irritability, and muscle tone dysregulation). Abnormalities in neurologic and systemic findings include roving eye movements (slow, conjugate, side-to-side, or circular movements of the eyes), hypotonia, dystonia, respiratory distress, anemia, failure to gain weight, abdominal distention, and poor feeding. A history of fetal distress and microcephaly might be associated. Partial or complete response of seizures to pyridoxine (PN) or pyridoxal 5′- phosphate (PLP) is reported, with seizure recurrence following vitamin B6 discontinuation. Mitochondrial encephalopathy-like presentation with lactic acidemia is possible. In six atypical cases with PDE-PLPBP described until now, the onset of different types of seizures (including epileptic spasms) is late, after the first month of life, and is associated with movement disorder, opistotonus, oculogyric crises, and partial response to ASMs (16).

Pyridoxine-dependent epilepsy due to pyridoxine 5′-phosphate oxidase (PNPO) deficiency is characterized by low plasma and cerebrospinal fluid levels of pyridoxine phosphate with sustained low activity of PLP-dependent enzymes (17). Two phenotypes are recognized: the classic form and the late-onset form. The classic form of the disease is most frequent (90%) and is characterized by seizure or status epilepticus onset in the first few days to 2 weeks of life, even *in utero*. Late-onset form of disease is a rare PNPO phenotype (10%) and is characterized by seizure onset after the neonatal period. Perinatal history might be eventful with data about premature birth, fetal distress, and low Apgar score at birth. A seizure semiology included myoclonic, clonic, or tonic seizures resistant to common ASMs, with response to life-long treatment with a B6 vitamer: pyridoxal 5′-phosphate in about 60% pts. and pyridoxine (PN) in 40%. Developmental delay with affected speech, cognition, and behavior is observed in 60% of the patients, while severe developmental delay is more likely in prolonged delay of specific treatment introduction (17).

Pyridoxine-responsive epilepsies are characterized by clinical findings suggestive of pyridoxine-dependent epilepsy, but without molecular confirmation of any of the three pyridoxine-dependent epilepsies. An increasing number of different neurologic and metabolic disorders associated with PRE have been reported: developmental and epileptic encephalopathies due to pathogenic variants in *CACNA1A*, *KCNQ2*, hyperprolinemia type II, molybdenum cofactor deficiency, hypophosphatasia, and hyperphosphatasia (1, 18, 19).

The aim of our study is to evaluate the etiology, phenotype, and long-term outcome in children with pyridoxine-responsive seizures, including children with PDE and PRE, and to define predictors of outcome.

## 2 Materials and methods

The study included all children with seizures with a response to pyridoxine treated in our hospital in the period from 2006 to 2025. All patients were divided into two groups: those with pyridoxine-dependent epilepsy (PDE) (I group) and those with pyridoxine-responsive epilepsy (PRE) (II group). Pyridoxine-dependent epilepsy is associated with pathogenic variants in *ALDH7A1, PNPO, and PLPBP* genes. Pyridoxine-responsive epilepsy (PRE, II group) is characterized by clinical and/or EEG response of the seizures resistant to at least two adequately selected and administered ASMs (phenytoin or carbamazepine are mandatory), but without molecular confirmation of any of the three pyridoxine-dependent epilepsies. Newborns with benign familial and non-familial neonatal seizures were diagnosed according to clinical presentation, family history, response to phenytoin/carbamazepine, and presence of the most frequent molecular finding of pathogenic variants of the *KCNQ2/3* genes, which was not included since they have a good prognosis.

Serial video electroencephalography (EEG), brain MRI, metabolic, genetic analyses (exome sequencing), and psychological assessment were done in all cases. Diagnosis of PDE was established according to suggestive clinical findings and biallelic pathogenic/likely pathogenic variants in *ALDH7A1, PNPO, PLPBP.* Supportive metabolic analyses, such as alpha-aminoadipic semialdehyde (*α*-AASA) concentration in urine and/or plasma and pipecolic acid concentration in plasma and CSF, or pyridoxamine concentration in CSF and plasma, were done sporadically since the analyses are not available in our country. Diagnosis of PRE was done according to clinical and/or EEG response to pyridoxine in the cases resistant to at least two adequately selected and administered ASMs. For standard video EEG recording, electrodes were placed according to the International 10–20 system of placement as recommended by the IFCN. The frequency filters used during recording were a low-pass filter of 70 Hz and a high-pass filter of 1 Hz. In all cases,- EEG was recorded before and after pyridoxine treatment, and electrographic improvement was included for pyridoxine efficacy assessment. The early-onset seizures were initially treated by antiseizure medication (ASM), and if at least two of the ASMs failed to stop the seizures, pyridoxine was introduced. Pyridoxine was initially given 100 mg/day IV for 2 consecutive days, followed by 20–30 mg/kg/day orally up to the cumulative 500 mg before assessment of pyridoxine efficacy. In a few cases with encephalopathy and status epilepticus, pyridoxine was given in intravenous infusion during the 5 consecutive days at a dosage of 100 mg/day. Positive pyridoxine response is defined if seizure stopping clinically and/or electrographically within the period of reaching a cumulative dosage of 500 mg of pyridoxine. Analyzed parameters were as follows: age of seizure onset, period from seizure onset to pyridoxine introduction, brain MRI, type of seizures initially and during the course of disease, etiology, additional ASM requirement, and predictors for outcome. Outcome included seizure control and the presence of developmental delay at the end of the follow-up. Patients were stratified into three groups according to timing of pyridoxine administration: within the first week, between the second week and 28th day, and after 28 days. Outcomes developmental delay, seizure freedom, and ASM requirements, were compared across groups by means of Fisher’s exact test. The Cochran–Armitage trend test was performed to evaluate for a monotonic trend in proportions across ordered groups. Mann–Whitney test, Fisher’s exact test, and Firth penalized logistic regression were used to test for statistical significance.

## 3 Results

A total of 21 patients were included: 10 with PDE (group I) associated with pathogenic variants *ALDH7A1* (7 cases), *PNPO* gene (2 cases), and *PLPBP* (1 case), and 11 patients with PRE (group II). The etiology of PRE included cerebrovascular insult (1 case), prematurity and hypoxic–ischemic encephalopathy (4 cases), developmental and epileptic encephalopathy due to pathogenic variants in SLC13A5 genes (2 cases), and unknown etiology with normal brain MRI and genetic findings (4 cases) (Table 1). In four cases with unknown etiology of PRE, benign familial and non-familial neonatal seizures were excluded, while interictal EEG showed burst, a suppression pattern in three cases, and depression of background activity in one.

**Table 1:** Etiology in patients divided into two groups: first group with pyridoxine-dependent epilepsy, and second with pyridoxine-responsive epilepsy.

Group of patients	No of patients	Etiology	No of patients
I group Pyridoxine-dependent epilepsy	10	*ALDH7A1*	7
		*PNPO*	2
		*PNPBP*	1
II group Pyridoxine-responsive epilepsy	11	Prematurity, HIE, negative genetics	4
		*SLC13A5*	2
		CVI in utero	1
		Unknown (negative MRI & genetics)	4

HIE, hypoxic ischemic encephalopathy; CVI, cerebrovascular insult.

In most of the patients, the seizure onset was within the first week of life, except one girl with atypical, late-onset PDE. Median age at seizure onset in both cohorts was 2 days (range 1–6 days for I group and 1–11 days for II group). Mann–Whitney U test demonstrated no significant difference in age at seizure onset between the two groups (*p* = 0.79). The median time from seizure onset to pyridoxine treatment was 12 days in patients with PDE (range 1–61 days) and 10 days (range 1–98) in patients with PRE. This difference was not shown to be statistically significant (*p* = 0.65). In all cases, the seizures stopped within 2 days of pyridoxine introduction. In the first group, prematurity is less frequent, only in one boy with PNPO, while in the second group, 4 of 11 patients were preterm newborns.

**Figure 1:**
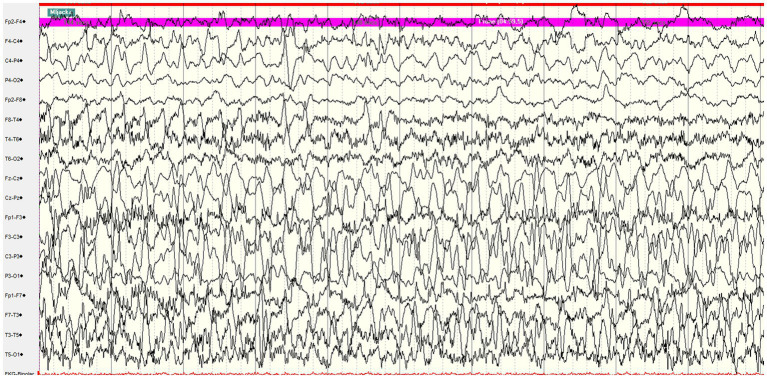
Ictal EEG in a boy before introducing pyridoxine during epilepsia partialis continua of the right hand and smacking, showing epileptic discharges of 3 Hz spike-waves above the left frontocentral region with spreading to the whole left hemisphere.

**Figure 2:**
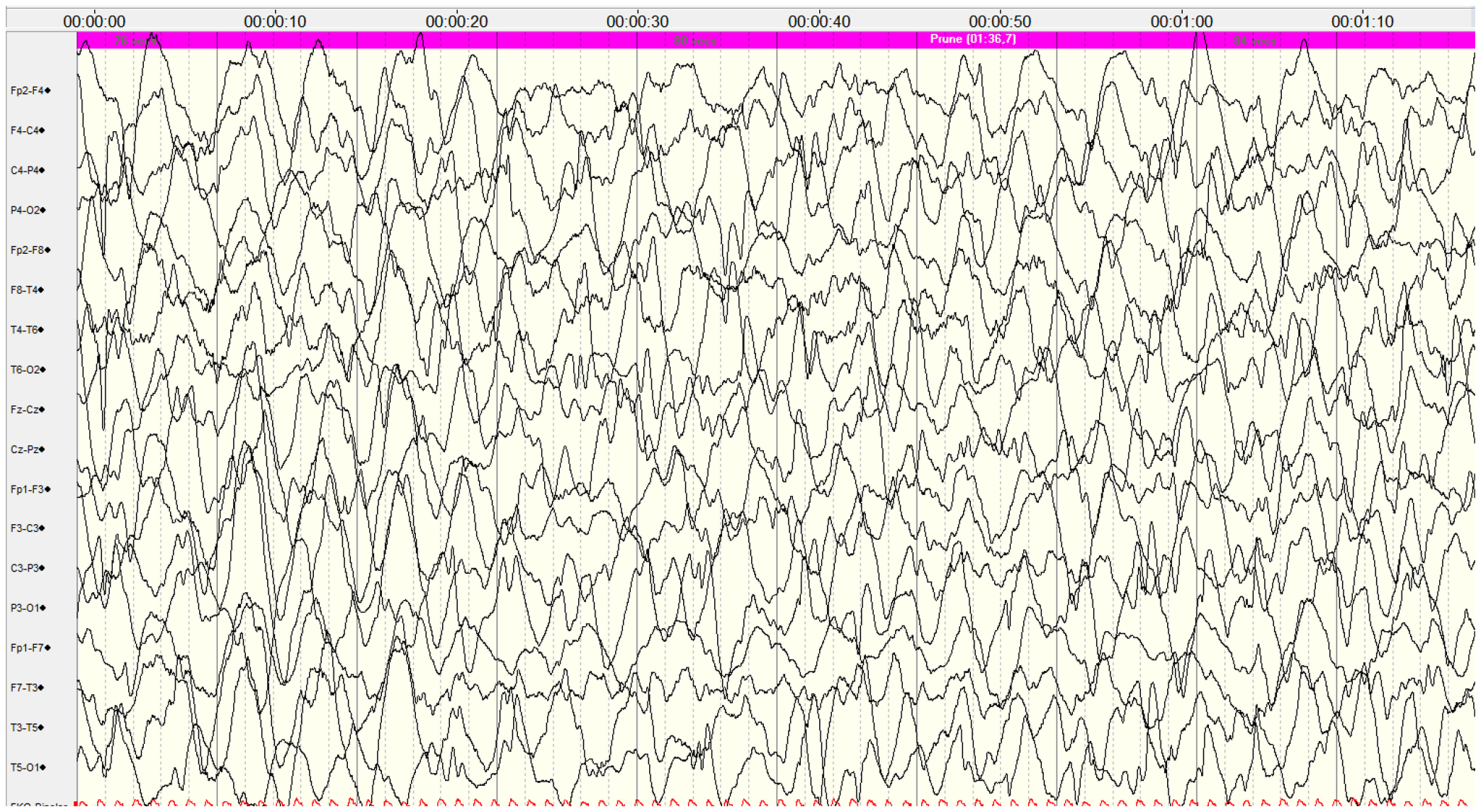
Electroencephalogram during non-convulsive status epilepticus (unresponsiveness, staring around, non-verbal) in a girl with late-onset PDE due to a pathogenic variant in ALDH7A1.

**Figure 3:**
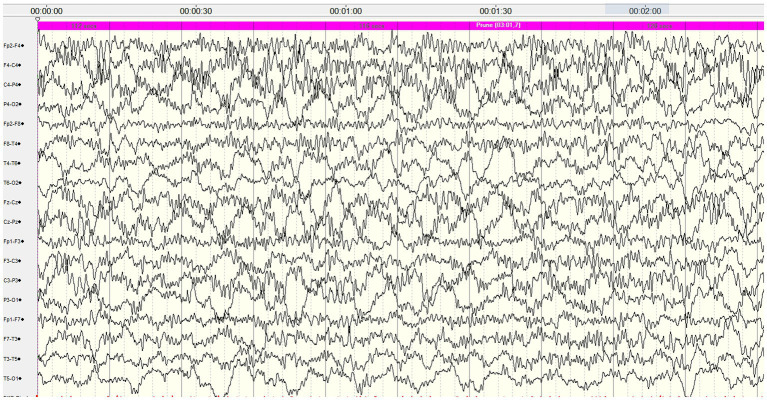
Electroencephalogram after intravenous administration of midazolam showed diffuse beta activity, while clinically non-convulsive status epilepticus was stopped in a girl with late-onset PDE due to a pathogenic variant in ALDH7A1.

Ictal EEG was recorded during 13 clinical seizures. During four multifocal clonic and myoclonic seizures, the EEG showed multifocal and generalized discharges (spike-waves, polyspikes, and sharp-waves). During eight focal seizures, the EEG recorded focal epileptic discharges commonly involving a whole hemisphere, presenting as rhythmic discharges of slow delta, theta, or alpha activity, or as high amplitude spikes-waves/sharp-waves. Ictal EEG during epilepsia partialis continua is presented in Figure 1. Ictal EEG during non-convulsive status epilepticus in a girl with an atypical form of PDE is presented in Figure 2, while midazolam response is presented in Figure 3. In one case, an electroencephalographic seizure was recorded as focal repetitive rhythmic activity lasting more than 30 s.

Developmental delay was present in 6/10 (60%) patients in the pyridoxine-dependent group and 5/11 (45.5%) patients in the pyridoxine-responder group. Seizure freedom was attained in 8/10 (80%) patients in the pyridoxine-dependent group and 8/11 (72.7%) patients in the pyridoxine-responder group. MRI abnormalities were seen in 8/10 (80%) patients in the pyridoxine-dependent group and 7/11 (63.6%) in the pyridoxine-responder group. The difference in these parameters between the two groups was not found to be significant.

### 3.1 Patients with pyridoxine-dependent epilepsy

The group of PDE included nine homozygote or compound heterozygote patients for pathogenic variants in the *ALDH7A1* (7 patients) and *PNPO* gene (2 patients). One boy with a typical clinical presentation of early onset refractory focal status epilepticus lasting 12 h (left side jerking, blinking, and eye deviation toward the right side), good response to pyridoxine, and brain MRI abnormalities was a heterozygote for *PLPBP* (clinical exome sequencing), while whole genome sequencing is in progress since it has recently become available in our country. He is classified in our cohort as PDE due to incomplete genetic results. Initial clinical presentation, such as types of seizure, presence of encephalopathy and status epilepticus, interictal electroencephalographic (EEG) findings, and initial treatment, is shown in Table 2.

**Table 2:** The age of seizure onset, type of initial seizures, presence of encephalopathy, interictal EEG, way of pyridoxine application, and period from seizure onset to pyridoxine treatment in children with pyridoxine-dependent epilepsy (group I).

Patient number genetic type	Age of seizure onset (day)	Type of initial seizures	Encephalopathy	Interictal EEG before PI	Pyridoxine (way of application)	Period from seizure onset to PI (day)
1. *ALDH7A1*	6	Focal, multifocal clonic, myoclonic status epilepticus	Hyperexcitability, opistotonus, sepsis-like, apnea, resp. distress	BS	I. V, stopped after 2nd infusion due to apnea, restart on 21st day of life	1
2. *ALDH7A1*	1	Focal clonic, myoclonic	Hyperexcitability, crying, movement disorder	Normal	I. V.	30
3. *ALDH7A1*	2	Focal, multifocal clonic	Movement disorders hyperexcitability	BS	I. V.	4
4. *ALDH7A1*	4	Focal clonic, EPC, myoclonic	Extreme hypotonia, sedation	Focal and multifocal ED	P. O.	61
5. *ALDH7A1*	1	Multifocal, myoclonic, bilateral clonic, cyanosis	Hyperexcitability, eyes deviation	Multifocal ED	I. V.	23
6. *ALDH7A1*	2	Focal clonic, multifocal, NCSE	Somnolence respiratory impairment	Multifocal ED	IV, interrupted due to respiratory impairment and infection	10
7. *ALDH7A1* Atypical form	120	Infantile spasms, focal	No	Multifocal ED	P. O.	580
8. *PNPO*	1	Serial focal, bilateral clonic, eyes twitching, chewing, status epilepticus lasting 45 min	Vomiting, hyper excitability	Multifocal ED	IV	12
9. *PNPO*	3	Focal, multifocal, myoclonic, lasting > 30 min	Hyperexcitability	BS	IV	17
10. *PLPBP*	4	Focal, left leg and arm jerking, status epilepticus lasting 12 h, blinking, eyes deviation toward right side	Movement disorders—uncontrolled excessive movements of the limbs	BS	I. V.	1

BS, burst-suppression pattern; ED, epileptic discharges; EEG, electroencephalogram; EPC, epilepsia partialis continua; IV, intravenous; NCSE, non-convulsive status epilepticus; PO, per os; PI, pyridoxine introduction.

One patient with *ALDH7A1* pathogenic variants had a typical form with late-onset seizures and an initial good response to ASMs. The first suspected focal jerks started at the age of 2 months, but they were very rare and not recognized as a seizure. At the age of 4 months, infantile spasms and focal seizures started. The patient showed partial response to a combination of ASMs (valproate, levetiracetam, phenobarbital, clobasam, and lacozamide). At the age of two and a half years, the girl started to experience recurrent episodes of refractory convulsive and non-convulsive status epilepticus (NCSE) with transient response to intravenous midazolam. Figure 2 showed ictal EEG during NCSE, while Figure 3 showed EEG 2 min after midazolam administration associated with clinical recovery of consciousness. This is the first time that pyridoxine was introduced in this girl, with a recommendation to continue the treatment until the results of genetic analyses. During the further 2 years of follow-up, the girl was seizure-free with withdrawal of ASMs and with the treatment with pyridoxine in a dosage of 30 mg/kg/d.

In the first group of patients, the median age of seizure onset was 2 days, in the range from 1 to 6 days, except one girl with a late onset seizure at the age of 2 months (60 days). The period from the first seizure to pyridoxine introduction was in the range from 1 to 61 days (median 12 days), except for the girl with late-onset seizure, where this period lasted 580 days.

The most frequent types of initial seizures were focal clonic (9 pts) and multifocal clonic (5 pts) seizures, while four patients had myoclonic jerks. In all cases, ictal and interictal EEG showed epileptic discharges, except one case with a normal interictal EEG with normal background activity without epileptic discharges (patient number 2 in Tables 2, 3). Six patients experienced status epilepticus: four convulsive status epilepticus, one epilepsia partialis continua (Figure 1), and one non-convulsive status epilepticus. In all cases, status epilepticus was stopped by 100 mg of pyridoxine IV infusion, after failure of at least two anticonvulsive drugs (phenobarbital, phenytoin, midazolam, or levetiracetam). Along with seizures, nine patients had encephalopathy presenting from depression of consciousness to hyperexcitability, prolonged crying, and motor agitation. In all children with status epilepticus and serial seizures, pyridoxine was given in intravenous infusion, while in only two children, the treatment was initiated with oral intake of pyridoxine. In two cases, intravenous infusion was interrupted due to apnea during the infusion, and in the other due to respiratory impairment, infection was associated with somnolence.

**Table 3:** Type of seizures during the course of disease, presence of developmental delay, and neuroimaging findings in children with pyridoxine-dependent epilepsy (group I).

Patient number genetic type	Type of the seizures during the course	Development delay	Neuroimaging brain MRI	Seizure freedom at the end of follow up	Follow up (years)
1. *ALDH7A1*	Rare focal induced by infection	Yes	Hydrocephalus, white matter reduction	Yes	16
2. *ALDH7A1*	No seizure	No	Megacysterna magna, no abnormalities in brain parenchyma	Yes	6
3. *ALDH7A1*	Focal	Yes	Ventrculomegaly, chronic hypoxic perithalamic lesions, Dandy-Walker	No	6
4. *ALDH7A1*	Focal, multifocal	Yes	Corpus callosum dysgenesis, white matter atrophy	Yes	3
5. *ALDH7A1*	No seizure	No	No abnormalities in brain parenchyma	Yes	13
6. *ALDH7A1*	No seizure	Yes, microcephaly	No abnormalities in brain parenchyma	Yes	2
7. *ALDH7A1* Atypical form	Focal, bilateral clonic, non-convulsive status epilepticus	Yes	Cystic PVL, cerebral white matter atrophy	Yes	3
8. *PNPO*	Infantile spasms, focal, generalized tonic–clonic	Yes	White matter hyperintensity	No	18
9. *PNPO*	During an infection occipital focal seizures At the age of 4y unprovoked gelastic seizure with bilateral tonic clonic.jerks	No	Posterior region gliosis	Yes	12
10. *PLPBP*	No seizure	No	Frontal white matter reduction, corpus callosum dysgenesis	Yes	9

PVL, periventricular leukomalatia.

We encountered two cases with PNPO deficiency, both with onset during the first days of life (first and third day), presenting as status epilepticus. Both children with PNPO deficiency were treated with pyridoxine in maintaining dosage of 30 mg/kg/day, with a recommendation to increase up to 45 mg/kg/day during the infection since pyridoxal phosphate is not available.

The first child is a compound heterozygote for pathogenic variants in the *PNPO* gene (c.529C > T (p. Arg177Ter),c.674G > A (p. Arg225His)). He experienced the first seizure on the third day of life, presenting as status epilepticus lasting more than 30 min of focal, multifocal, and myoclonic jerks. Pyridoxine, given intravenously on the 17th day after seizure onset, was successful in stopping the seizure. During the 12-year follow-up period, rarely occipital focal seizures with visual impairment were observed dominantly during infection. At the age of 4 years, the boy experienced a rare gelastic seizure. With a recommendation to increase the dosage of pyridoxine from 30 up to 45 mg/kg/day during the infection, no recurrence of seizure was observed. Brain MRI showed posterior region gliosis. The boy had normal development and academic achievement.

The second case is a girl with PDE due to *PNPO* pathogenic variants with a history of infantile spasms during the course of the disease. She is the second child in a family with a history that the first child had died due to neurological complications defined as “perinatal asphyxia.” She experienced the first seizures within the first day of life as serial focal jerks with prolonged duration in the form of status epilepticus (SE) and bilateral spreading of the jerks, associated with blinking and chewing. Since parenteral phenobarbital and midazolam in continuous infusion failed, pyridoxine was given in intravenous infusion, and the response was prompt after the first infusion of 100 mg of pyridoxine. The girl was seizure-free for 2 months with the oral intake of pyridoxine. At the age of 2 months, flexion infantile spasms started, followed by focal and bilateral clonic seizures. Vigabatrine was added, and the dosage of pyridoxine was increased up to 30 mg/kg/d. This treatment was effective partially because during the long-term follow-up of 18 years, the girl continued to experience focal and generalized tonic–clonic seizures with frequency up to 10 per month. Besides pyridoxine, the girl was treated by levetiracetam, clobasam, valproate, carbamazepine, phenobarbital, oxcarbazepine, lamotrigine, and clonazepam. Brain MRI showed diffuse white matter abnormalities, and developmental delay was observed. Currently, the girl reported paroxysmal episodes of discomfort as “butterflies from the stomach going up” associated with visual failure lasting less than 1 min.

In nine of 10 cases with PDE, interictal EEG before pyridoxine introduction showed pathological findings, while in one case, interictal EEG was normal. Interictal EEG recorded multifocal epileptic discharges in five patients, and a burst-suppression pattern in four patients.

During the course of the disease, seizures were recurring in six patients, in all as focal motor seizures, in two with bilateral spreading, and one child was suffering infantile spasms with early onset in the second month of life. In two cases, recurrent seizures were provoked by infection before they started to practice increasing the dosage of pyridoxine during the infection. Various brain abnormalities revealed by MRI are detailed in Table 3, along with data on disease outcomes. At the end of the follow-up (mean duration 8.2 years, range 2–18 years), in 80% seizure, freedom was achieved.

### 3.2 Patients with pyridoxine-responsive epilepsy

A group of patients with pyridoxine-responsive epilepsy included 11 patients with various etiologies (Table 1). Median age at seizure onset was 2 days (range 1–11 days), while the median time from seizure onset to pyridoxine treatment was 10 days (range 1–98). In five patients, the first seizures were focal motor, in six multifocal, and in one associated with myoclonic jerks. In contrast to the patients with PDE, where almost all patients (9/10) had initial encephalopathy, in the group of PRE, only 3/11 had encephalopathy, three were somnolent, one was hyper-excited, while 5/11 were without encephalopathy. Almost half of the cases (5/11) with PRE were preterm newborns. The data about initial manifestation and treatment in patients with PRE are presented in Table 4.

**Table 4:** The age of seizure onset, type of initial seizures, presence of encephalopathy and prematurity, interictal EEG, way of pyridoxine application, and period from seizure onset to pyridoxine treatment in children with pyridoxine-responsive epilepsy (group II).

Patient number	Age of seizure onset (day)	Type of initial seizures	Encephalopathy	Interictal EEG before PI	Pyridoxine (way of application)	Prematurity	Period from seizure onset to PI (day)
1	1	Focal	No	BS	P. O.	No	2
2	11	Multifocal	Somnolence, encephalopathy	BS	I. V.	Yes	5
3	1	Focal	No	BS	I. V.	No	14
4	2	Multifocal	No	BA depression	I. V.	No	18
5	3	Multifocal	Somnolence	Multifocal ED	I. V.	Yes	98
6	2	Focal	No	BS	P. O.	Yes	29
7	9	Focal	Hemiparesis, somnolence	Multifocal ED	I. V.	No	10
8	3	Multifocal, myoclonic	Hyperexcitability	Multifocal ED	I. V.	Yes	2
9	5	Focal	Encephalopathy	BS	I. V.	Yes	1
10	1	Multifocal	No	Multifocal ED	I. V.	No	24
11	2	Multifocal	Encephalopathy	BS	I. V.	No	1

BA, background activity; BS, burst-suppression pattern; ED, epileptic discharges; EEG, electroencephalogram; IV, intravenous; NCSE, non-convulsive status epilepticus; PO, per os; PI, pyridoxine introduction.

Interictal EEG before pyridoxine introduction showed burst-suppression pattern in six cases, multifocal epileptic discharges in four cases, and depression of background activity in one.

During the course of the disease, recurrent seizures were experienced by three cases: two had focal seizures with bilateral spreading, and one had a combination of infantile spasms and focal seizures. Two cases with developmental and epileptic encephalopathy caused by pathogenic variant *SLC13A5* continued to suffer seizures after initial response to pyridoxine, requiring additional ASMs (patients number 6 and 10 in Tables 4, 5). MRI findings are presented in Table 5, along with data about the seizures and disease outcome. During the follow-up period (mean 6 years, range 2–16 years), developmental delay was observed in 5/11, and seizure freedom was achieved in 8/11.

**Table 5:** Type of seizures during the course of disease, presence of developmental delays, and neuroimaging findings in children with pyridoxine-responsive epilepsy (group II).

Patient number	Type of the seizures during the course	Development delay	Neuroimaging brain MRI	Seizure freedom at the end of follow up	Follow up (years)
1.	No seizure	No	White matter atrophy frontal bilateral	Yes	4
2.	Infantile spasms, focal	Yes	Hypoxic and ischemic changes	No	4
3.	No seizure	No	Normal	Yes	11
4.	No seizure	No	Normal	Yes	4
5.	No seizure	No	Ventriculomegaly supratentorial hypoxic changes	Yes	4
6.	Focal, bilateral clonic	Yes	Normal	No	4
7.	No seizure	No	White matter hiperintensity, thin corpus callosum	Yes	16
8.	No seizure	No	White matter atrophy frontotemporal bilateral	Yes	3
9.	No seizure	Yes	Hypoxic and ischemic changes SAH	Yes	2
10.	Focal, bilateral clonic	Yes	White matter atrophy frontal bilateral	No	2
11.	No seizure	Yes	Normal	Yes	12

SAH, subarachnoid hemorrhage.

### 3.3 Outcome

Outcome was assessed at the end of the follow-up period and included the presence of developmental delay and achieving seizure freedom. Developmental delay was present in 6/10 (60%) patients in the pyridoxine-dependent group and 5/11 (45.5%) patients in the pyridoxine-responder group. Seizure freedom was attained in 8/10 (80%) patients in the pyridoxine-dependent group and 8/11 (72.7%) patients in the pyridoxine-responder group.

#### 3.3.1 Predictors of outcome in patients with pyridoxine-dependent epilepsy

To evaluate the impact of treatment initiation on the outcome, the patients were stratified into three groups according to timing of pyridoxine administration: within the first week, between the second week and 28th day, and after 28 days. Developmental delay was observed in 67% (2/3) of PDE patients treated within the first week, 75% (3/4) of those treated between 1e week and 1 month, and 100% (2/2) of those treated after 1 month. Seizure freedom was achieved in 67% (2/3), 50% (2/4), and 50% (1/2) of patients across the three groups, respectively. Ongoing ASM requirement was present in 67% (2/3), 50% (2/4), and 50% (1/2) of patients across the three groups, respectively. For all three outcomes, no statistically significant difference was observed (Fisher’s *p* > 0.05), and no significant trends were identified (Cochran-Armitage *p* > 0.05). One patient with the atypical presentation of PDE was excluded from these analyses because of late-onset seizures. The results are presented in Table 6.

**Table 6:** The outcome in PDE patients stratified into three groups depending on treatment timing.

Outcome	First week (*n* = 3)	1 week–1 month (*n* = 4)	>1 month (*n* = 2)	Fisher’s exact *p*-value	Trend test p-value
Developmental delay, N (%)	2 (67%)	3 (75%)	2 (100%)	>0.05	>0.05
Seizure freedom, N (%)	2 (67%)	2 (50%)	1 (50%)	>0.05	>0.05
Ongoing ASM requirement, N (%)	2 (67%)	2 (50%)	1 (50%)	>0.05	>0.05

ASM, antiseizure medication.

A clinically relevant difference was observed in the requirement for additional ASM. Additional ASMs were required in 4/8 patients with abnormal MRI findings, whereas none of the patients without structural abnormalities required treatment other than pyridoxine (Fisher’s exact test *p* = 0.47). Although not statistically significant, this absolute difference suggests a potential association. Stratified analysis by lysine-restricted dietary treatment revealed that none of the patients receiving a lysine-restricted diet required additional ASMs, regardless of MRI findings (0/3). In contrast, among patients not receiving dietary treatment, additional ASM was required only in those with abnormal MRI findings (4/6 vs. 0/1; Fisher’s exact test *p* = 0.43). This suggests that dietary therapy may modify the relationship between MRI abnormalities and ASM requirement.

Developmental delay was more frequently observed in patients with abnormal MRI findings (6/8 vs. 0/2; Fisher’s exact test *p* = 0.13). Similarly, multiple seizure types were observed only in patients with abnormal MRI findings (3/8 vs. 0/2; Fisher’s exact test *p* = 1.00), although no statistically significant association was detected.

Seizure freedom rates were comparable between groups (6/8 vs. 2/2; Fisher’s exact test *p* = 1.00), with no evidence of a meaningful association with MRI findings. Overall, these findings are limited by the small sample size and should be interpreted with caution. Penalized logistic regression using Firth’s method was performed to account for sparse data and zero cell counts; however, the resulting estimates were imprecise with wide confidence intervals.

#### 3.3.2 Predictors of outcome in patients with pyridoxine-responsive epilepsy

In the pyridoxine-responder cohort, the median time from seizure onset to pyridoxine initiation was 6 days (range 1–98) in patients who were seizure-free, and 24 days (range 1–29) in patients who were not seizure-free. Patients who showed developmental delay were started on pyridoxine treatment at a median of 5 days after seizure onset (range 1–29), while patients who later had no evidence of developmental delay had pyridoxine treatment initiated at a median time of 12 days (range 2–98). None of these differences was shown to be significant. Similarly, as in the patients with PDE, earlier onset of seizure and duration of median period from seizure onset to initiation of pyridoxine are not predictors for developmental delay and seizure recurrence.

In contrast to the pyridoxine-dependent cohort, no meaningful associations were observed between MRI findings and clinical outcomes in the responder group. The requirement for additional ASM was similar between patients with abnormal and normal MRI findings (2/7 vs. 1/4; Fisher’s exact test *p* = 1.00). The same can be said for developmental delay (3/7 vs. 2/4; *p* = 1.00), seizure freedom (5/7 vs. 3/4; *p* = 1.00), and the presence of multiple seizure types (3/7 vs. 1/4; *p* = 1.00); these were comparable between groups. Penalized logistic regression yielded odds ratios close to unity with wide confidence intervals, further indicating no evidence of association. These findings suggest that, within this subgroup, MRI abnormalities do not appear to be associated with clinical outcomes.

## 4 Discussion

Pyridoxine-responsive seizures are part of two main categories: one is pyridoxine-dependent epilepsy, and the other is pyridoxine-responsive epilepsy. Pyridoxine-dependent epilepsy is characterized by seizures uncontrolled by ASMs, but responsive clinically and electrographically to large daily supplements of pyridoxine, and with molecular genetics confirmation of any of three known genotypes. In pyridoxine-responsive epilepsy, the findings are suggestive of pyridoxine-dependent epilepsy, but without molecular confirmation of one of three PDE genotypes (14, 19). In our cohort of 21 patients, there was almost an equal frequency of PDE (10pts) and PRE (11pts) collected during the long period of time, nearly two decades. Among our patients with PDE, the most frequent etiology was antiquitin deficiency (pathogenic *ALDH7A1* variants), in seven cases. This finding is in correlation with the literature report that antiquitin deficiency is the most frequent cause of PDE (1, 3). Typically, the onset of seizure is during the first weeks, but atypical presentations are possible, as we described in one of our patients, an onset at the age of 4 months with infantile spasms and with partial response to ASMs. We presented that benzodiazepines might be effective for stopping non-convulsive status epilepticus in the atypical form of PDE- ALDH7A1. Two of our patients with PDE (one PDE- ALDH7A1, one PDE-PLPO) experienced infantile spasms at an early age of 2 and 4 months. In one case with PDE-ALDH7A1, diagnosis was established with a delay, and the girl was only on ASMs for 2 years before pyridoxine treatment. So, we suggest a pyridoxine trial in all infants with the onset of infantile spasms, particularly if the onset is at an early age.

Common MRI findings in patients with PDE include midline brain abnormalities, particularly agenesis or hypoplasia of the corpus callosum, cerebral atrophy, and white matter abnormalities. Enlarged cisterna magna and ventriculomegaly (5). Similarly, we found white matter brain MRI abnormalities in six of 10 patients, corpus callosum dysgenesis in one patient, ventriculomegaly in two cases, megacysterna magna in one and Dandy-Walker in one case.

In the group of 11 PRE, the etiology was heterogeneous, including premature neonates with hypoxic ischemic encephalopathy, *SLC13A5*-associated developmental and epileptic encephalopathies, and unknown etiology with negative MRI and genetics.

The results showed no significant difference between the two cohorts, PDE and PRE, in terms of median age at seizure onset, clinical presentation of seizures (focal and multifocal were most frequent in both groups), frequency of brain abnormalities, and median time from seizure onset to pyridoxine treatment. It means that accordingly clinical presentation of seizure and time of onset is not easy to distinguish PDE from PRE. Our results supported the opinion of Baxter, who reported that pyridoxine dependency is rare, and since atypical presentations are relatively frequent, a trial of pyridoxine is recommended in all cases of early-onset intractable seizures or status epilepticus, whatever the suspected cause (18, 19).

Some differences were observed, and they might be helpful for clinicians in determining the differences between the two groups. Prematurity was more frequent in patients with PRE than in PDE (only in one boy with PNPO). Encephalopathy is a prominent clinical finding for PDE (in nine of 10), while only 3/11 with PRE had encephalopathy. Encephalopathy in PDE contributes to a “dramatic” initial presentation of disease (16), and we also recognized that almost all patients with PDE had hyperexcitability, hyperalertness, inconsolable crying, jitteriness, and irritability. In patients with PRE, encephalopathy was rare and dominantly presented as depression of motor activity and somnolence.

According to the guidelines and consensus-based recommendations from a special report from the ILAE Task Force on Neonatal Seizures (1), a trial of pyridoxine (add-on to ASM) is recommended in newborns with clinical and EEG features related to PDE, and newborns with retractable seizures were unresponsive to second-line ASM without an identified etiology (1). Our investigation contributes with evidence that even in newborns with known etiology of refractory seizures, such as hypoxic ischemic encephalopathy, developmental and epileptic encephalopathies other than PDE, a trial of pyridoxine might be successful and recommended.

Despite adequate treatment with pyridoxine, in our cohort, developmental delay was present in 60% patients with PDE, and 45.5% patients with PRE, suggesting that pyridoxine is sufficient to stop the seizures, but could not completely prevent developmental delay, and that other underlying mechanisms contribute to delay in neurological development and seizure outcome.

Several pathogenic mechanisms of seizures and developmental delays in PDE-ALDH7A1 have been proposed, and most of them are related to pyridoxal-5′-phosphate deficiency: elevated *α*- AASA and P6C in the central nervous system; pyridoxal-5′-phosphate deficiency for intracellular biochemical reactions in the human body beside using pyridoxine; abnormalities in metabolism of a main cerebral inhibitory neurotransmitter—gamma aminobutyric acid (GABA); decreased production of final product of lysine metabolism—acetyl-coenzyme A, due to α-AASA dehydrogenase deficiency; and decreased production of succinate—the end product of GABA metabolism (6–8).

Early and adequate treatment is essential in children with PDE (1, 20). Outcomes (developmental delay, seizure freedom, and ASMs requirement), were compared across three stratified groups of PDE patients according to timing of pyridoxine administration: within the first week, between the second week and 28th day, and after 28 days. The results of our study showed that PDE patients with late pyridoxine introduction, more than 1 week after seizure onset, had an increasing frequency of developmental delay and less common seizure freedom achievement. For all three outcomes, no statistically significant difference and no significant trends in our PDE cohort might be explained by small sample size, heterogeneity, and confounding variables. Additionally, the results of our dataset showed that even in children with early treatment, the outcome might be variable in terms of developmental delay. That data support the opinion that the accumulation of neurotoxic intermediates and functional deficiency of pyridoxal-5′-phosphate in patients with PDE might start very early, even prenatally. Abnormalities in brain development, such as micropcephaly, white matter abnormalities, periventricular or temporal cysts, anomalies of the corpus callosum, and global brain underdevelopment with broad gyri and shallow sulci (15), and the appearance of seizures *in utero*, support prenatal brain affection in children with PDE. Prenatal treatment has raised much interest (21).

According to literature data, several pathogenic mechanisms of seizures and developmental delay in PDE-ALDH7A1 are supposed, and most of them are related to pyridoxal-5′-phosphate deficiency (6–8), but pathogenic mechanisms in other PDEs are still unclear. These results are in contrast to data from the literature that late onset seizures are predictors for a favorable outcome (20). The authors suggested that a favorable outcome in late-onset PDE might be explained by a combination of factors (20). In our cohort, seizure freedom was attained in 80% patients in the pyridoxine-dependent group and 72.7% patients in the pyridoxine responder group. The patients with MRI abnormalities required additional ASMs more frequently, and developmental delay was more frequently observed in patients with abnormal MRI findings but without statistical significance. In two of our patients, a recurrent seizure was provoked by infection. It is important to recommend to patients to increase, even duplicate, the dosage of pyridoxine during the infection.

In conclusion, etiologic heterogeneity and phenotype overlapping between pyridoxine-dependent and pyridoxine-responsive seizures, atypical presentations of PDE, and good initial response to pyridoxine regardless of genetic and neuroimaging findings, suggest introduction of pyridoxine in all neonates and infants when two ASMs have failed. Despite appropriate pyridoxine treatment, more than half of patients with PDE had developmental delays, and resistant seizures persisted in a certain percentage. None of the analyzed clinical parameters showed significant predictive value for outcome. This finding might suggest the complexity of the underlying mechanisms in PDE and PRE. The limitation of our research was a small sample size, which resulted in the study being underpowered. Therefore, further international trials and collaboration between scientists and clinicians are essential to clarify some of those issues, and to improve the outcome in patients with pyridoxine-dependent and responsive epilepsies.

## Data Availability

The raw data supporting the conclusions of this article will be made available by the authors, without undue reservation.
